# Modeling the effect of exposure notification and non-pharmaceutical interventions on COVID-19 transmission in Washington state

**DOI:** 10.1038/s41746-021-00422-7

**Published:** 2021-03-12

**Authors:** Matthew Abueg, Robert Hinch, Neo Wu, Luyang Liu, William Probert, Austin Wu, Paul Eastham, Yusef Shafi, Matt Rosencrantz, Michael Dikovsky, Zhao Cheng, Anel Nurtay, Lucie Abeler-Dörner, David Bonsall, Michael V. McConnell, Shawn O’Banion, Christophe Fraser

**Affiliations:** 1 Google Research, Mountain View, CA USA; 2 Nuffield Department of Medicine, University of Oxford, Oxford, UK; 3 Department of Medicine, Stanford University School of Medicine, Stanford, CA USA

**Keywords:** Epidemiology, Health policy

## Abstract

Contact tracing is increasingly used to combat COVID-19, and digital implementations are now being deployed, many based on Apple and Google’s Exposure Notification System. These systems utilize non-traditional smartphone-based technology, presenting challenges in understanding possible outcomes. In this work, we create individual-based models of three Washington state counties to explore how digital exposure notifications combined with other non-pharmaceutical interventions influence COVID-19 disease spread under various adoption, compliance, and mobility scenarios. In a model with 15% participation, we found that exposure notification could reduce infections and deaths by approximately 8% and 6% and could effectively complement traditional contact tracing. We believe this can provide health authorities in Washington state and beyond with guidance on how exposure notification can complement traditional interventions to suppress the spread of COVID-19.

## Introduction

The COVID-19 pandemic has brought about tremendous societal and economic consequences across the globe, and many areas remain deeply affected. Due to the urgency and severity of the crisis, the poorly understood long-term consequences of the virus, and the lack of certainty about which control measures will be effective, many approaches to stopping or slowing the virus are being explored. In seeking solutions to this problem, many technology-based non-pharmaceutical interventions have been considered and deployed^1^, including data aggregation to track the spread of the disease, GPS-enabled quarantine enforcement, AI-based clinical management, and many others.

Contact tracing, driven by interviews of infected persons to reveal their interactions with others, has been a staple of epidemiology and public health for the past two centuries^2^. These human-driven methods have been brought to bear against COVID-19 since its emergence, with some success^3^. Unfortunately, owing in part to the rapid and often asymptomatic spread of the virus, these efforts have not been successful in preventing a global pandemic. Further, as infections have reached into the millions, traditional contact tracing resources have been overwhelmed in many areas^4,5^. Given these major challenges to traditional contact tracing, it has been suggested that apps that make use of Bluetooth technology can assist in detecting exposures to those carrying the virus, and serve as a complementary tool to human contact tracing initiatives^6^.

Technological solutions in this space have never been deployed at scale before, and their effectiveness is unknown. There is an acute need to understand their potential impact, to establish and optimize their behavior as they are deployed, and to harmonize them with traditional contact tracing efforts. Specifically, we will examine these issues in the context of the Exposure Notification System (ENS), developed by Apple and Google, which is currently being adopted by many states and countries^7^. In this system, GPS and location data are not used—instead, Bluetooth alone is utilized to exchange anonymous, randomly-generated IDs which can later be checked against a list of positive cases. In order to protect user privacy and build user trust, ENS does not require users and their contacts to be identified or located, and recognition of each user’s exposure risk level can take place only on the user’s smartphone^8^.

To improve our understanding of this new approach, we employ individual-based computational models, also known as agent-based models, which allow the exploration of disease dynamics in the presence of complex human interactions, social networks, and interventions^9,10^. This technique has been used to successfully model the spread of Ebola in Africa^11^, malaria in Kenya^12^, and influenza-like illness in several regions^13,14^, among many others. In the case of COVID-19, the OpenABM-Covid19 model by Hinch et al.^15^ has been used to explore smartphone-based interventions in the United Kingdom. Individual-based models simulate individuals and their interactions in home, work, and community contexts, using epidemiological parameters to guide the compartments in an expanded SEIR model^16^ and demographic parameters to simulate individuals and their interactions. Although past work has studied disease transmission^17–19^, progression^20^, and social distancing interventions^10,21,22^, we seek to understand the combined effect of exposure notifications and non-pharmaceutical interventions in an environment calibrated to the demographics^23^, occupational structure, and epidemic trend of that location.

In this work, we adapt the OpenABM-Covid19 model to simulate the ENS approach, and apply it to data from Washington state in the United States to explore possible outcomes. We use data at the county level to match the population, demographic, and occupational structure of the region, and calibrate the model with epidemiological data from Washington state and Google’s Community Mobility Reports for a time-varying infection rate^24^. Similar to Hinch et al., we find that digital exposure notification can effectively reduce infections, hospitalizations, and deaths from COVID-19, even if just roughly 15% of the overall population participates. We extend the findings by Hinch et al. to show how digital exposure notification can be deployed concurrently with traditional contact tracing and social distancing to suppress the current epidemic and aid in various “reopening” scenarios. We believe the demographic and occupational realism of the model and its results have important implications for the public health of Washington state and other health authorities around the world working to combat COVID-19.

## Results

### Digital exposure notification

We present forward-looking simulations for Washington state counties by comparing multiple hypothetical scenarios with combinations of digital exposure notification, manual contact tracing, and social distancing. Each simulation uses the same calibrated model up to July 11, 2020, at which point the hypothetical interventions are implemented. Beyond this date, each simulation uses the final calibration model parameters, except where explicitly specified as part of the intervention. For each simulated intervention we report the number of infections (daily and cumulative), cumulative number of deaths, number of hospitalizations, number of tests per day, and fraction of the population in quarantine. The simulation runs for a consistent 300 days from the beginning of our mobility data, March 1, 2020, through Dec 25, 2020, plus the additional calibrated seeding period before March 1. Unless otherwise stated, the reported result is the mean value over 10 runs with different random seeds of infection.

Results may be affected by the end date of the simulation because of the time it takes some interventions to have their full effect. We believe that a time horizon of approximately 5 and a half months is long enough to be practically useful for public health agencies who are considering deploying such interventions, but short enough to minimize the long-term uncertainty and effects of externalities such as a vaccine becoming available.

**Fig. 1:**
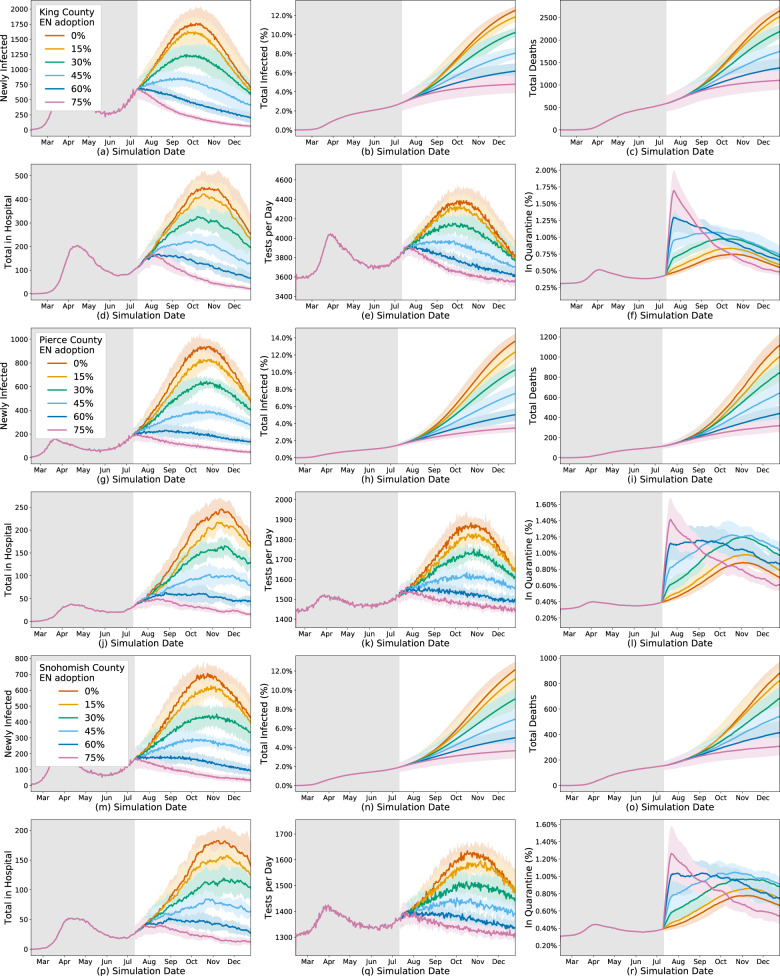
Simulation time series. Simulation results for various levels of exposure notification app uptake (among the total population) during 2020, with the app being implemented on July 11, 2020 in King County (**a**–**f**), Pierce County (**g**–**l**), and Snohomish County (**m**–**r**). The shaded areas represent one standard deviation. Plots (**a**, **g**, **m**) are the new infections on the given day, which consistently decrease with EN adoption, although the largest variances occur in the mid-range of adoption changes. Plots (**b**, **h**, **n**) are the total infected percentage, Plots (**c**, **i**, **o**) are the total deaths, Plots (**d**, **j**, **p**) are the total in hospital, all of which naturally vary by new infections given the natural progression through the compartmental model. Plots (**e**, **k**, **q**) are the total number of tests performed per day, which also varies by new infections as tests are only performed on symptoms, not on trace, so they correlate with the proportion of newly infected individuals who eventually show symptoms. Plots (**f**, **l**, **r**) are the percent of people in quarantine at that time step, which varies non-linearly with the infection rates due to increased quarantines from increased contact tracing but decreased quarantines with decreasing infection rates.

**Fig. 2:**
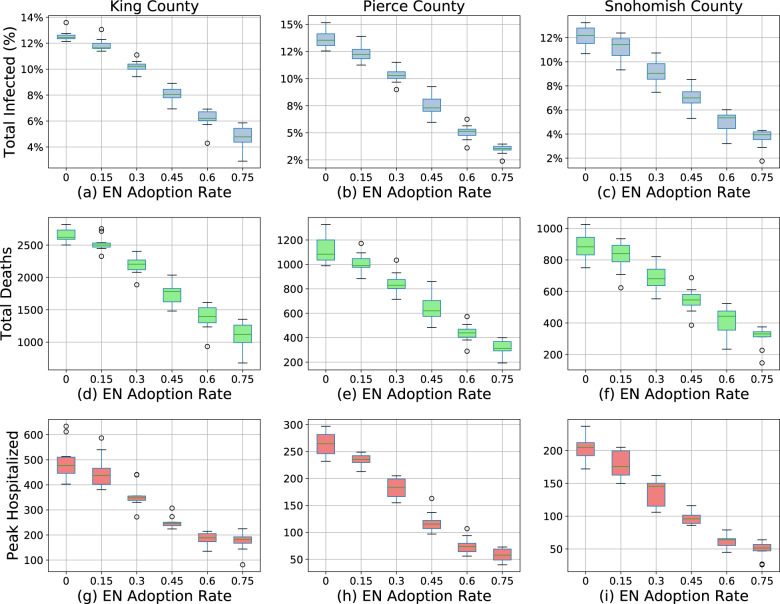
Peak metrics vs exposure notification adoption rates. Estimated total infected percentage (**a–c**), total deaths (**d**–**f**), and peak in hospital (**g**–**i**) (*y*-axes) of King (**a**, **d**, **g**), Pierce (**b**, **e**, **h**), and Snohomish (**c**, **f**, **i**) counties for various levels of exposure notification (EN) app uptake among the population (*x*-axis) between July 11, 2020 and December 25, 2020. The boxes represent the Q1 to Q3 quartile values with a line at the median. The whiskers show the range of the data (1.5 * (Q3–Q1)) and any outlier points are past the end of the whiskers.

We first study the effect of a digital exposure notification app at different levels of app adoption (15%, 30%, 45%, 60%, and 75%) of the population in each county. As a baseline, we compare those results to the “default” scenario assuming no change in behavior or interventions beyond July 11, 2020. The results show an overall benefit of digital exposure notification at every level of app adoption (Figs. 1, 2). When compared to the default scenario of only self-isolation due to symptoms, each scenario results in lower overall incidence, mortality, and hospitalizations. Unsurprisingly, the effect on the epidemic is more significant at higher levels of app adoption. An app with 75% adoption reduces the total number of infections by 56–73%, 73–79%, and 67–81% and deaths by 52–70%, 69–78%, and 63–78% for King, Pierce, and Snohomish counties, respectively. However, even at a relatively low level of adoption of 15%, there are meaningful reductions in total infections of 3.9–5.8%, 8.1–9.6%, and 6.3–11.8% and total deaths of 2.2–6.6%, 11.2–11.3%, and 8.2–15.0% for King, Pierce, and Snohomish counties, respectively.

**Fig. 3:**
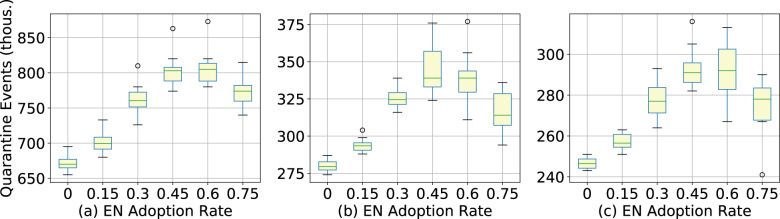
Quarantine events vs exposure notification adoption. Estimated total quarantine events of King (**a**), Pierce (**b**), and Snohomish (**c**) counties for various levels of exposure notification app uptake among the population from July 11, 2020 to December 25, 2020. Note that even for the “default” (0% exposure notification app uptake) scenario there is a non-zero number of quarantine events because this assumes that symptomatic and confirmed COVID-19 positive individuals will self-quarantine at a rate of 80%, even in the absence of an app.

In addition to its ability to suppress the epidemic, we also evaluate the effects of exposure notification adoption on the total number of quarantine events. There is an incentive to minimize the quarantine rate because of the perceived economic and social consequences of stay-at-home orders. At 15% exposure notification adoption the total number of quarantine events increases by 4.6–6.4%, 6.6–6.8%, and 5.8–10.2% for King, Pierce, and Snohomish counties (Fig. 3). In general, the higher the level of exposure notification adoption the greater the number of total quarantine events, with the exception of very high levels of adoption (60% and 75%) where this number plateaus or even decreases, likely due to the significant effect of the intervention in suppressing the overall epidemic in those scenarios.

### Manual contact tracing

Next, we study the potential impact of manual contact tracing in suppressing the epidemic as a function of the contact tracing workforce size. We refer to guidance by the Office of the Governor of WA State with a minimum recommendation of 15 tracers per 100,000 people as well as the staffing rates for King County including all available staffers (105 FTE for 2.253 million people, or 4.7 per 100,000) and the National Association of County & City Health Officials (NACCHO) recommended staffing levels during epidemics of 30 staff per 100,000 people^25^. We set the tracing delay to one day, which is the optimistic estimate for time to contact trace, as the goal for Washington state is to notify 80% of contacts within 48 hours^26^. We similarly use the King County Phase 2 Application to compute the expected number of initial contact tracing interviews as well as follow-up notifications. Over a two-week period, 22 staff members contacted 336 individuals for initial interviews and 941 for close contact notifications, or approximately 1 initial interview and 3 notifications per day per staff member.

**Fig. 4:**
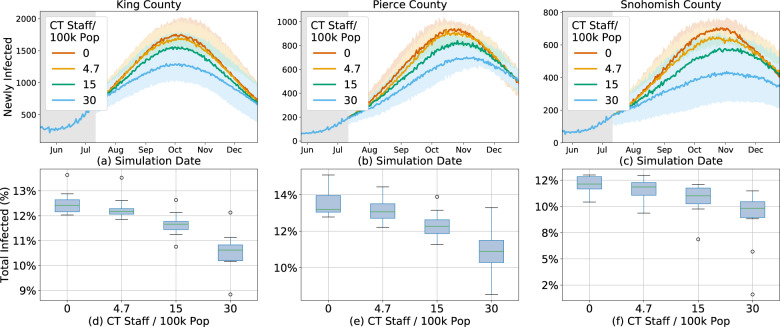
Infections under manual contact tracing. Estimated effect of manual contact tracing on new infections (**a**–**c**) and total infected percentage (**d**–**f**) at various staffing levels per 100k people in King (**a**, **d**), Pierce (**b**, **e**), and Snohomish (**c**, **f**) counties between July 11, 2020 and December 25, 2020.

Manual tracing with the full desired staffing levels of 15 workers per 100,000 people is able to affect the epidemic trend in all three counties, but has a significantly smaller effect at current staffing levels (Fig. 4). Unsurprisingly, the impact for a given level of staffing is dependent upon the current epidemic trend, reinforcing the need for concurrent interventions to effectively manage the epidemic.

Additionally, we compare the performance of exposure notification to manual contact tracing to (1) establish similarities between relative staffing level and exposure notification adoption and (2) to verify an additive effect of concurrent manual tracing and exposure notification.

**Fig. 5:**
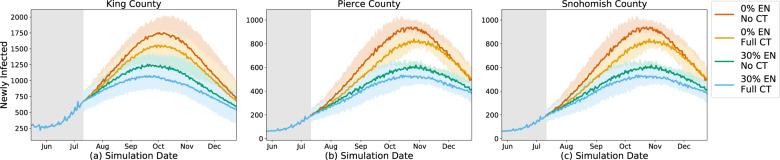
Combined effects of manual contact tracing and exposure notification system. Comparison between manual contact tracing (CT) at the recommended staffing level and exposure notification (EN) at 30% adoption in King (**a**), Pierce (**b**), and Snohomish (**c**) counties. In all three counties, the combined effect is greater than individual contributions by either system.

We see improvements in all cases when combining interventions (Fig. 5). In all three counties, exposure notification has a stronger effect at the given staffing and adoption levels, but adding either intervention to the other results in reduced infections, albeit to different extents based on the trend of the epidemic. This suggests that both methods are useful separately and combined, and that the trend affects the relative utility of the interventions.

### Concurrent interventions under behavioral changes

While the results shown above suggest that the interventions are effective in suppressing the COVID-19 epidemic to various degrees, in practice, health organizations will implement multiple intervention strategies simultaneously to try to curb the spread of the virus while also allowing controlled reopenings. Therefore, we also study the combined effect of concurrent interventions including digital exposure notification, manual contact tracing, and network interaction changes. We explore changes to social distancing in Supplementary Figs. 1, 2.

We examine the effects of combined NPIs under various “reopening” scenarios by increasing the number of interactions in every interaction network, including households, workplaces, schools, and random networks. Specifically, we increase these interactions by a given percentage from the levels as of July 11, 2020 (0% reopen) up to the levels at the very start of the epidemic before broad-based mobility reductions (100% reopen). Given the average number of interactions *i* for network *n* at the end of the baseline as *i*_*b,n*_ and before the lockdown as *i*_0,*n*_, the network reopening percentage *p* (in 0–100%) defines the current relative interactions under reopening *i*_*c,n*_ as

1 $$i_{c,n} = \frac{p}{{100}}\left( {1 - i_{b,n}} \right) + i_{b,n}.$$

**Fig. 6:**
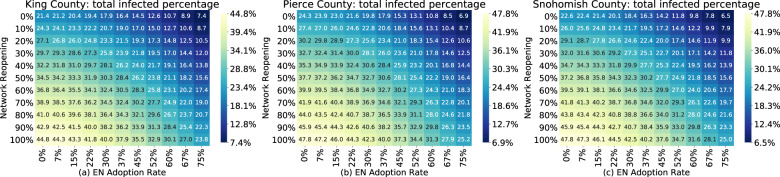
Simulated reopenings under varied exposure notification adoption rates. Estimated total infected percentage as of December 25, 2020 as a function of simultaneous network reopening and exposure notification app adoption rates, assuming fully staffed manual contact tracing (15 workers per 100,000 people), in King (**a**), Pierce (**b**), and Snohomish (**c**) counties. The infectious rate increases due to a 10–20% reopening are balanced by decreases due to a 22–37% exposure notification app adoption, although the effect varies by county.

The infectious rate increases due to a 10–20% reopening are balanced by decreases due to a 22-37% exposure notification app adoption, although the effect varies by county (Fig. 6). This shows that limited additional reopenings may be possible after introducing exposure notification alongside existing fully staffed manual tracing (15 staff per 100,000 people), but that social distancing remains an important measure under these circumstances. Additionally, there is an increased effect to adding exposure notification under greater reopening scenarios.

As part of the Washington State Department of Health’s “Safe Start” plan, a key target metric to reopen Washington is to reach fewer than 25 new cases per 100,000 inhabitants over the prior two weeks^26^. We examine how many days it would take to reach that target under the combined NPIs. With the spike in cases at the end of the baseline, the trajectory for reaching these targets without renewed lockdowns is out of the range of the simulations. Therefore, to show the relative benefits of the NPIs, we introduce an artificial renewed lockdown at the mobility levels averaged over the month before the Phase 2 reopenings (Phase 1.5 for King County) that occurred on June 5th. Using this averaged mobility from May 6 to June 5, we model the relative effects of manual tracing and exposure notification on the Washington Safe Start key metric.

**Fig. 7:**
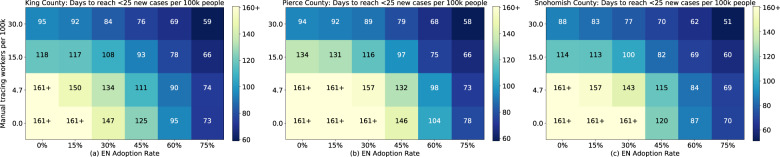
Days to key metric under varied intervention levels. Estimated number of days from July 11, 2020 for King, Pierce, and Snohomish counties to reach the Washington state goal of fewer than 25 new cases per 100,000 people over the trailing 14 days, as a function of manual tracing workforce capacity and exposure notification app adoption, given a renewed lockdown to the average level over the month before June 5th. Simulated results shown for King (**a**), Pierce (**b**), and Snohomish (**c**) counties show the ability of the interventions to suppress the epidemic by the primary reopening metric.

We find that, for all three counties, the recommended staffing levels of manual tracing and moderate exposure notification adoption rates can provide a meaningful reduction in the amount of time it takes to achieve this metric (Fig. 7). Under the recommended standard for manual tracing, adding exposure notification at 30% adoption results in reaching the target in 92%, 87%, and 85% of the time versus no exposure notification for King, Pierce, and Snohomish counties respectively. At the reduced levels of 4.7 tracers per 100,000 population, the target is reached in less than 83% and 88% of the time for King and Snohomish, respectively, although the exact ratio can not be calculated as the metric is not achieved in the baseline simulation.

## Discussion

We conducted a model-based estimation of the potential impact of a digital ENS in Washington state by extending the OpenABM-Covid19 simulation framework. We calibrated our model using real-world data on human mobility and accurately matched epidemiological data in Washington state’s three largest counties.

Similar to Hinch et al.’s report on digital contact tracing in the UK^15^, we found that exposure notification can meaningfully reduce infections, deaths, and hospitalizations in these Washington state counties at all levels of app uptake, even if a small fraction of the population participates. We showed how exposure notification can be combined with manual contact tracing to further suppress the epidemic, even if the two interventions do not explicitly coordinate. Our simulations showed that the simultaneous deployment of both interventions can help these counties meet a key incidence metric defined by the Safe Start Washington plan. The potential overall effect of exposure notification seems to be greater than even optimal levels of manual contact tracing, likely because of its ability to scale and better identify random interactions.

We found that quarantine rates, which contribute to the social and economic cost of these interventions, do not strictly increase with exposure notification adoption. In some cases, fewer people are quarantined even though a greater fraction of the population participates in the app, which we attribute to successful suppression of the epidemic at high levels of exposure notification app adoption. Given a longer simulation time horizon we may see a similar effect even at the lower levels of exposure notification adoption.

Finally, we looked at the combined effects of exposure notification and manual tracing in the context of different reopening scenarios, where mobility and interaction levels increase to the pre-epidemic levels. Our results suggest that both interventions are helpful in counterbalancing the effect of reopening, but are not sufficient to offset new cases except at very high levels of adoption and manual tracing staffing. As a result, we believe that continued social distancing and limiting person-to-person interactions is essential. Future work is needed to study targeted reopening strategies, such as reopening specific occupation sectors or schools, or more stringent social distancing interventions in places that do reopen.

While we have attempted to add realistic elements and calibrate it with the best available data, choices and simplifications made surrounding the behavior of the individuals, their movements in the world, in particular a lack of cross-county movement, disease dynamics, and many others, mean that the results should be viewed as an exploration of possible outcomes, not a prediction^27^. We plan to explore the effects of cross-county movement in our future work.

We simulate a two-day wait from symptom onset to COVID-19 test result receipt and acknowledge this as a key assumption underlying our findings. Ferretti et al.^28^ showed that such delays have a significant impact on the intervention’s efficacy. Rapid testing protocols can shorten this delay and are essential for epidemic control^15^.

We use a high RT-PCR test specificity of 0.999, which limits the effects of removing individuals from the network due to false positives rather than actual infections and provides a conservative estimate of the effects of contact tracing. Lowering this value could have a small effect on the epidemic trajectory, but possibly a greater effect on the perception of utility and efficacy of the system. For future modeling work studying a more accurate overall characterization of quarantine rates, predictive value, or public perception, specificity should be set closer to an average of the most recent findings in the range of 0.97–0.992^29,30^.

We used published COVID-19 mortality data to calibrate model parameters. While arguably a good proxy for true infection numbers, the published mortality data can be scarce and noisy, especially in small counties, resulting in potential difficulties of modeling with accuracy.

The synthetic occupation networks are based on the latest employment data corresponding to the fourth quarter in 2019^31^. Since the beginning of the pandemic, the size and structure of occupation networks may have significantly changed compared to the latest available data.

In our work, we used the mobility data along with a changepoint to model time-varying infection rates. While the changepoint vector models the net effect of various latent factors, it may be limited when multiple change points or more complex latent factors exist. The changepoint rate is homogeneously distributed to the random network and occupational networks, which may vary heterogeneously in reality.

For future work, we consider coordination between different regions when deploying exposure notification as part of a suite of non-pharmaceutical interventions. The United States has seen a highly spatially varied response to the COVID-19 pandemic, with significant consequences to epidemic control^32^. Under varying cross-county and cross-state flows, we seek to quantify the empirical efficiency gap between coordinated and uncoordinated deployments and policies around testing, tracing, and isolation in which exposure notification can aid. The beginning of such collaborations is evident in the consortia of state governments such as the Western States Pact and a multi-state council in the northeast, both working together to coordinate their responses. We expect that coordinated deployments of digital exposure notification applications and public policies may lead to more effective epidemic control as well as more efficient use of limited testing and isolation resources.

## Methods

### Modeling individual interactions and COVID-19 epidemiology

**Fig. 8:**
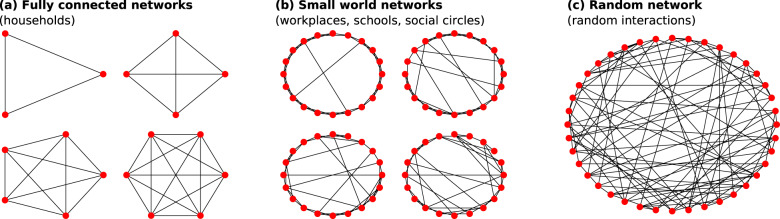
Example interaction networks. Examples of fully connected (**a**), Watts–Strogatz small-world (**b**), and random (**c**) networks that define interactions among synthetic agents in households (**a**), workplaces, schools, social circles (**b**), and random (**c**) settings.

To model the combined effect of digital exposure notification and other non-pharmaceutical interventions (NPIs) in Washington state, we use a model first proposed by Hinch et al.^15^, who have also made their code available as open source on GitHub^33^. OpenABM-Covid19 is an individual-based model that models interactions of synthetic individuals in different types of networks based on the expected type of interaction (Fig. 8). Workplaces, schools, and social environments are modeled as Watts–Strogatz small-world networks, households are modeled as separate fully connected networks, and random interactions, such as those on public transportation, are modeled in a random network. The networks are parameterized such that the average number of interactions matches the age-stratified data in ref. ^34^. Contacts between synthetic individuals in those interaction networks have the potential for transmission of the virus that causes COVID-19 and are later recalled for contact tracing and possible quarantine.

While the original model by Hinch et al.^33^ included a single occupation network for working adults, we extend this to support multiple networks for workplace heterogeneity. This is motivated by increasing evidence that workplace characteristics play an important role in the spread of SARS-CoV-2, such as having to work in close physical proximity to other coworkers and interacting with the public. Baker et al. found that certain U.S. working sectors experience a high rate of SARS-CoV-2 exposure, including healthcare workers, protective services (e.g., police officers), personal care and services (e.g., child care workers), community, and social services (e.g., probation officers)^35^. As another example, the Centers for Disease Control and Prevention (CDC) has issued specific guidance to meat and poultry processing workers due to the possible increased exposure risk in those environments^36^. As a result, we model individual industry sectors separately so that each sector has its own small-world network and is parameterized with real-world data on its size and interaction levels.

In OpenABM-Covid19, transmission between infected and susceptible individuals through a contact is determined by several factors, including the duration since infection, susceptibility of the recipient (a function of age), and the type of network where it occurred (home networks assume a higher risk of transmission due to the longer duration and close proximity of the exposure). Individuals progress through stages of susceptible, infected, recovered, or deceased. In this model, the dynamics of progression through these stages are governed by several epidemiological parameters, such as the incubation period, disease severity by age, asymptomatic rate, and hospitalization rate, and are based on the current literature of COVID-19 epidemiology. We reference the complete list of the epidemiological parameters^37^ and any modifications to those are described in the subsequent sections and listed in Supplementary Table 1.

### Modeling Washington state

In this work, we model the three largest counties in Washington state—King, Pierce, and Snohomish—with separate and representative synthetic populations. The demographic and household structure were based on data from the 2010 U.S. Census of Population and Housing^38^ and the 2012–2016 ACS Public Use Microdata Sample^39^. We combined Census and Public Use Microdata Sample (PUMS) data using a method inspired by^40^. For each Census block in Washington state, we took distributions over age, sex, and housing type from several marginal tables (called Census Summary tables) and from the PUMS, and combined them into a multiway table using the iterative proportional fitting (IPF) algorithm. We then resampled the households from the PUMS to match the probabilities in the multiway table. The resulting synthetic population in each Census block respects the household structure given by PUMS and matches marginals from the Census Summary tables.

Our synthetic working population was drawn to match the county-level industry sector statistics reported by the U.S. Bureau of Labor Statistics in their Quarterly Census of Employment and Wages for the fourth quarter of 2019^31^. We also used a report by the Washington State Department of Health (DOH) containing the employment information of lab-confirmed COVID-19 cases among Washington residents as of May 27, 2020 to parameterize each occupation sector network^41^ (See Supplementary Fig. 3 for the breakdown of cases by occupation sector). For each sector, we use its lab-confirmed case number weighted by the total employment size as a multiplier factor to adjust the number of work interactions of that occupational network. While the DOH report does not explicitly measure exposure risk for different industries, it is, to the best of our knowledge, the best source of data for confirmed COVID-19 cases and occupations to date. Our model should be refined with better data from future work that studies the causal effect of workplace characteristics on COVID-19 transmission. A complete list of the occupation sectors and interaction multipliers can be found in Supplementary Tables 2, 3.

### Modeling interventions

In the OpenABM-Covid19 model, if an individual presents with COVID-19 symptoms, they receive a test and are 80% likely to enter a voluntary 7-day isolation with a 2% drop out rate each day for noncompliance. If the individual receives a positive test result, they isolate for a full 14 days from initial exposure with a daily drop out rate of 1%. Prior to confirmation of the COVID-19 case via a test result, the household members of the voluntarily self-isolating symptomatic individual do not isolate, which is in line with current recommendations by the CDC^42^. Household quarantines may still occur through digital exposure notification or manual contact tracing, described in the following sections. We set the test sensitivity at 80% for tests taken at least three days after infection, which is on the conservative end of the range reported by a systematic review of false-negative rates of RT-PCR tests for COVID-19^43^. Test specificity was set to 99.9%, which is also inline with real-world studies of RT-PCR false-positive rates^29,44^. Both sensitivity and specificity are set conservatively for the purposes of the interventions, which trigger exposure notifications and contact tracing on positive results. A higher specificity is therefore a more conservative estimate, since lower values would cause more people to be isolated from their network.

We simulate digital exposure notification in OpenABM-Covid19 by broadcasting exposure notifications to other users as soon as an app user either tests positive or is clinically diagnosed with COVID-19 during hospitalization. The model recalls the interaction networks of this app user, known as the “index case”, to determine their first-order contacts within the previous 10 days. Those notified contacts are then 90% likely to begin a quarantine until 14 days from initial exposure with a 2% drop out rate each day for noncompliance. See the repository^33^ for a more comprehensive description of the model.

While the actual ENS allows health authorities to configure notifications as a function of exposure distance and duration, our model does not have the required level of resolution and instead assumes that 80% of all “too close for too long” interactions are captured between users that have the app. (See Supplementary Figs. 4, 5 for a sensitivity analysis of this parameter).

The overall effect of digital exposure notification depends on a number of factors that we explore in this work, including the fraction of the population that adopts the app and the delay between infection and exposure notification. As an upper bound on app adoption, we configure the age-stratified smartphone population using data on smartphone ownership from the U.S. from the Pew Research Center^45^ for ages 20+ and Common Sense Media^46^ for ages 0–19. Since this data was not available for Washington state specifically we assumed that the U.S. distribution was representative of Washington state residents.

We introduce to OpenABM a new type of active intervention, which is traditional or “manual” contact tracing. In contrast to digital exposure notification, human tracers work directly with index cases to recall their contact history without the proximity detection of a digital app. Those contacts are then given the same quarantine instructions as those traced through the digital app. We configure the simulation such that manual contact tracers have a higher likelihood of tracing contacts in the household and workplace/school networks (100% and 80%, respectively) than for the additional random daily contacts (5%), based on the assumption that people will have better memory of and ability to identify contacts in the former (e.g., involving family members or coworkers) compared to the latter (e.g., a random contact at a restaurant). Additionally, we configure the capacity of the contact tracing workforce with parameters for workforce size, maximum number of index-case interviews per day, and maximum number of tracing notification calls per day following those interviews. Tracing is initiated on an index case after either a positive test or hospitalization, subject to the capacity in that area. Finally, we add a delay parameter between initiation of manual tracing and finally contacting the traced individuals to account for the processing and interview time of manual tracing.

### Model calibration

Model calibration is the process of adjusting selected model parameters such that the model’s outputs closely match real-world epidemiological data. To calibrate OpenABM-Covid19 for Washington state we use components of a Bayesian SEIR model by Liu et al.^47^ for modeling COVID-19. They extend the classic SEIR model by allowing the infection rate to vary as a function of human mobility and a latent changepoint to account for unobserved changes in human behavior. We fit that model to Washington state county-level mortality data from *The New York Times*^48^ and mobility data from the Community Mobility Reports published by Google and made publicly available^24^. The Community Mobility Reports are created with aggregated, anonymized sets of data from users who have turned on the Location History setting, which is off by default. No personally identifiable information, such as an individual’s location, contacts, or movement, is ever made available^49^. The reports chart movement trends over time by geography, across different categories of places such as retail and recreation, groceries and pharmacies, parks, transit stations, workplaces, and residential. We note that, because of the opt-in nature of this dataset, it may not be representative of the overall population.

We extend the methodology in Liu et al. to model calibration in OpenABM-Covid19 by applying the time-varying infection rate coefficients to the relevant county-specific parameters that guide user interaction levels and disease transmission likelihood. More specifically, the number of daily interactions in the random and occupation networks, *Ri*(*t*) and *Wi*(*t*), are scaled by the mobility coefficient, *m*(*t*) at time step *t*, which is calculated based on the aggregated and anonymized location visits from the Community Mobility Reports. The time-dependent infectious rate, *β*(*t*), is scaled by a weighting term, *σ*(*t*), which depends on how far time step *t* is from a learned changepoint, which is modeled as a negative sigmoid. Both *σ*(*t*) and *m*(*t*) are learned functions and are described in more detail in Liu et al.^47^ Supplementary Figs. 6–8 show the learned functions that are used to calibrate OpenABM-Covid19.

Finally, we use an exhaustive grid search to compute two OpenABM-Covid19 parameters for each county: its initial infectious rate and the infection seed date (from 3.0 to 7.0 for the infectious rate parameter and 35 day period for the infection seed date). The infectious rate is the mean number of individuals infected by each infectious individual with moderate-to-severe symptoms, and can be considered a function of population density and social mixing. The infection seed date is the date at which the county reaches 30 total infections, possibly before the first official cases due to asymptomatic and unreported cases. We pick the parameters where the simulated mortality best matches the actual COVID-19 mortality from epidemiological data, as measured by root-mean-square error (RMSE). See Supplementary Table 4 for the calibrated parameter values that were used for this study.

**Fig. 9:**
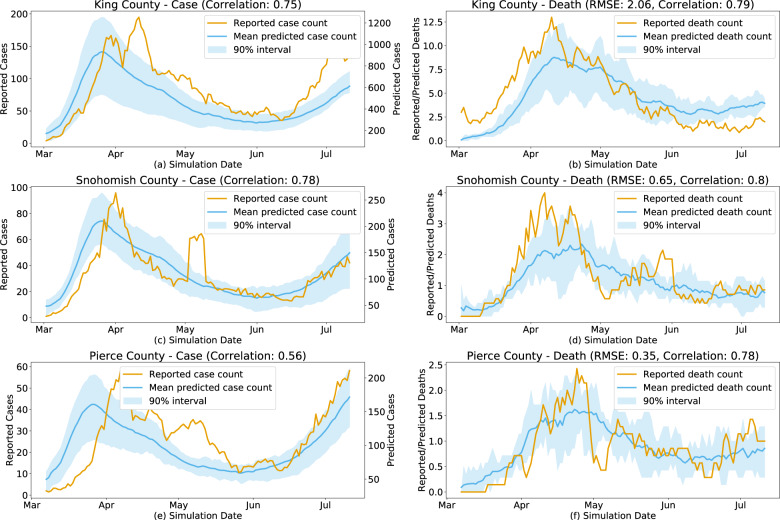
Calibrated simulation vs observed epidemic dynamics. Daily reported and predicted COVID-19 deaths in King County, WA (Correlation: 0.75) (**a**), Pierce County, WA (Correlation: 0.78) (**c**), and Snohomish County, WA (Correlation: 0.56) (**e**) and daily reported and predicted COVID-19 cases for King County, WA (RMSE: 2.06, Correlation: 0.79) (**b**), Pierce County, WA (RMSE: 0.65, Correlation: 0.80) (**d**), and Snohomish County, WA (RMSE: 0.35, Correlation: 0.78) (**f**).

The results of the calibrated models for King, Snohomish, and Pierce counties are shown in Fig. 9. Note that while there is a strong correlation in the predicted and reported incidence, the absolute predicted counts are approximately 6X higher than those that were officially reported. We attribute this difference to the fact that OpenABM-Covid19 is counting all asymptomatic and mild symptomatic cases that may not be recorded in reality. This is approximately consistent with the results of a seroprevalence study by the CDC that estimated that there were 6–24 times more infections than official case report data^50^.

### Reporting summary

Further information on research design is available in the Nature Research Reporting Summary linked to this article.

## Supplementary information

Supplementary Information

Reporting Summary

## Data Availability

The data used in this study are publicly available, including the “COVID-19 Community Mobility Reports” (https://www.google.com/covid19/mobility) and “Coronavirus in the U.S.: Latest Map and Case Count” (https://www.nytimes.com/interactive/2020/us/coronavirus-us-cases.html).

## Code availability

The code that supports the findings of this study is licensed under the GNU General Public License v3.0 and available at https://github.com/BDI-pathogens/OpenABM-Covid19.
