# Supplementary material for: Modeling the effect of exposure notification and non-pharmaceutical interventions on COVID-19 transmission in Washington state
Source: NPJ Digit Med. 2021 Mar 12;4:49. doi: 10.1038/s41746-021-00422-7 (PMC7955120; doi:10.1038/s41746-021-00422-7)
Supplement: Supplementary file 1 — Supplementary Information [file 41746_2021_422_MOESM1_ESM.pdf]

Supplementary Information

Further analysis of concurrent interventions

To better understand the effects of network reopenings, we plot additional primary metrics for a 50% network reopening and see significant reductions in nearly all metrics at even 30% adoption (Supplementary Fig. 1).

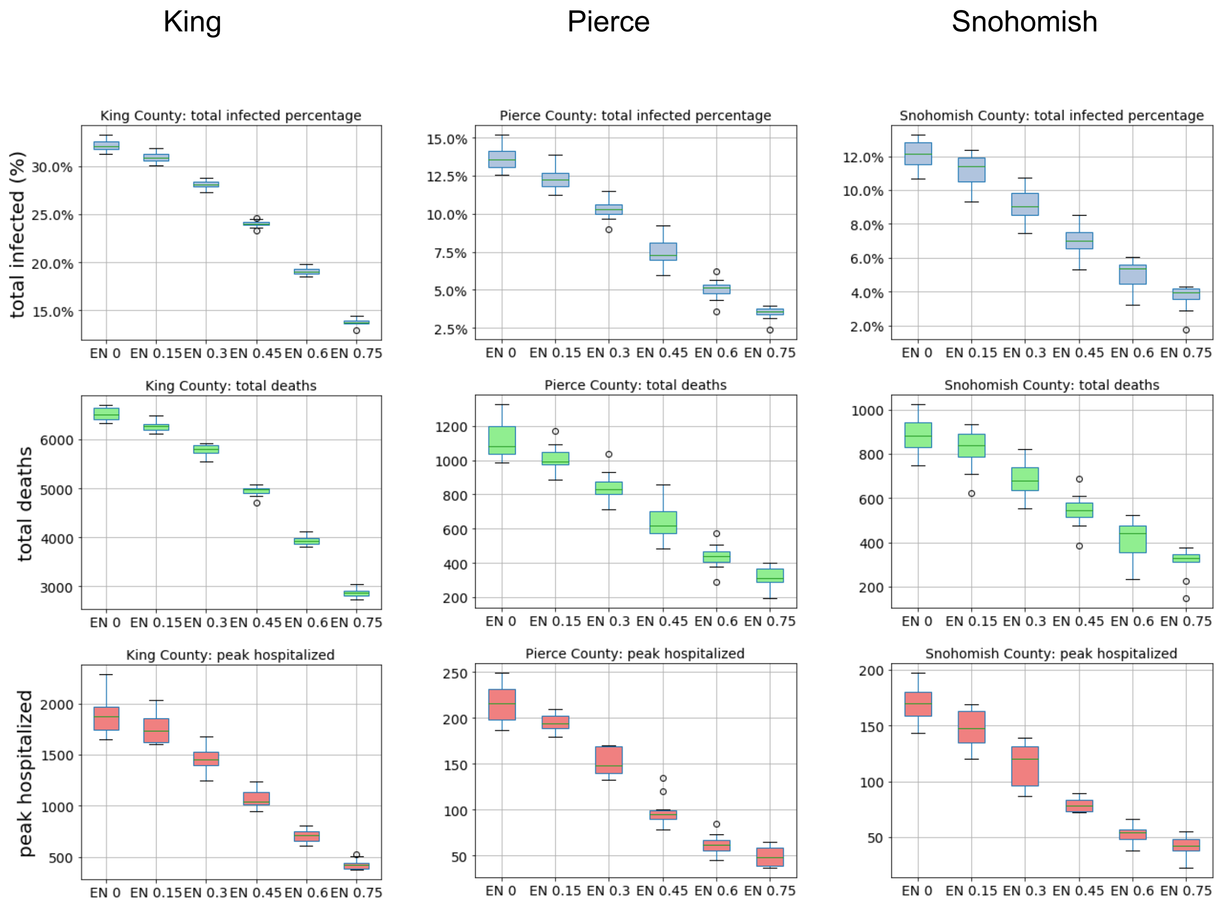

Supplementary Fig. 1. Estimated total infected percentage, total deaths, and peak hospitalized under a 50% reopening scenario (an increase of 50% of the difference between pre-lockdown and post-lockdown network interactions) at various exposure notification adoption rates for King,

Pierce, and Snohomish Counties, assuming no change to social distancing $\beta(t)$ after the baseline and 15 manual contact tracers per 100k people.

In additional to network reopenings, we model social distancing as the relative infectiousness of random and occupation network interactions, where increasing social distancing decreases the relative transmission on a network by a multiplicative factor relative to their initial values (i.e., before broad-based social distancing and mobility reductions). For example, social distancing of 1.7x is equivalent to multiplying the relative transmission by 1 / 1.7 = 0.6. Note that this does not change the number of individual interactions, but rather the likelihood of transmission of each individual encounter, for example, through mask usage, physical distancing, improved hygiene, personal protective equipment, etc. We display the results of social distancing vs exposure notification adoption rate in Supplementary Fig. 2.

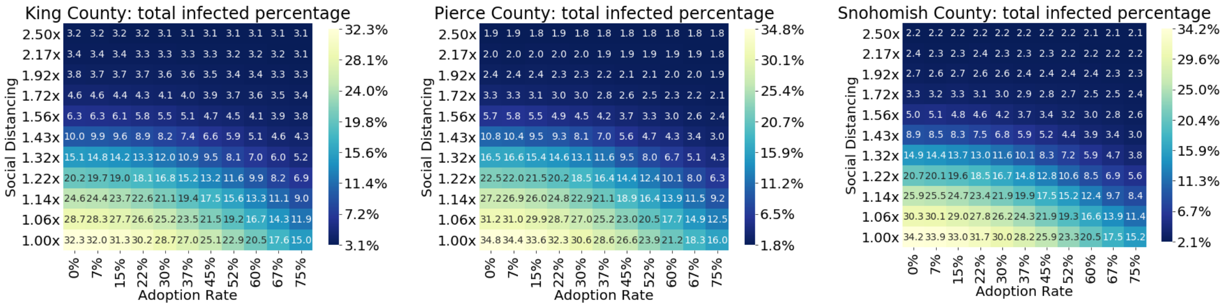

Supplementary Fig. 2. Estimated total infected percentages between July 11 to December 25, 2020 for King, Pierce, and Snohomish counties as a function of simultaneous social distancing and exposure notification app adoption. Social distancing is expressed as the infectiousness of random and occupation network interactions, relative to their initial values (i.e., before broad-based social distancing and mobility reductions).

Model parameters

	King County	Pierce County	Snohomish County
household_size_1	329,114	106,018	87,197
household_size_2	306,979	113,855	102,421
household_size_3	139,176	57,920	52,794
household_size_4	115,757	47,534	46,414
household_size_5	45,162	21,908	19,812
household_size_6	30,375	14,740	13,048
population_0_9	278,073	126,887	110,638
population_10_19	258,328	122,564	108,699
population_20_29	317,005	124,748	99,418
population_30_39	359,688	127,308	116,327
population_40_49	323,457	118,680	119,699
population_50_59	307,938	121,318	120,245
population_60_69	229,274	92,467	84,857
population_70_79	109,487	45,409	39,978
population_80	69,534	25,599	22,222
n_total	2,252,784	904,980	822,083
app_users_fraction_0_9	0.23		
app_users_fraction_10_19	0.75		
app_users_fraction_20_29	0.96		
app_users_fraction_30_39	0.92		
app_users_fraction_40_49	0.92		
app_users_fraction_50_59	0.79		
app_users_fraction_60_69	0.66		
app_users_fraction_70_79	0.53		
app_users_fraction_80	0.53		

Supplementary Table 1. The household (number of households with N person(s)), overall population, and smartphone population distribution for King, Pierce, and Snohomish counties.

Occupational sectors

NAICS Code	Sector Name	Employment Size*		
		King County	Pierce County	Snohomish County
11	Agriculture, Forestry, Fishing and Hunting	7,952	3,008	4,725
21	Mining	1,244	546	548
22	Utilities	5,813	1,916	351
23	Construction	226,711	72,603	72,510
31-33	Manufacturing	315,030	52,807	181,570
42	Wholesale Trade	188,971	39,099	27,194
44-45	Retail Trade	499,834	108,437	106,976
48-49	Transportation and Warehousing	156,542	54,590	14,446
51	Information	373,581	6,495	12,623
52	Finance and Insurance	126,708	25,076	28,694
53	Real Estate Rental and Leasing	88,850	15,908	9,819
54	Professional, Scientific, and Technical Services	399,492	31,437	40,091
55	Management of Companies and Enterprises	94,516	2,474	4,366
56	Administrative and Support and Waste Management and Remediation Services	223,083	67,857	44,478
61	Educational Services	73,614	14,574	6,370
62	Health Care and Social Assistance	476,480	153,035	102,666
71	Arts, Entertainment, and Recreation	79,193	14,771	11,224
72	Accommodation and Food Services	350,659	85,375	69,380
81	Other Services (except Public Administration)	144,458	33,609	22,195

* Employment size is estimated based on the employment data in 2019 Q4[2].

Supplementary Table 2. Sizes of occupational networks used for King, Pierce, and Snohomish counties.

NAICS Code	Sector Name	Adjustment factor
11	Agriculture, Forestry, Fishing and Hunting	2.0
21	Mining	1.0
22	Utilities	1.0
23	Construction	1.0
31-33	Manufacturing	1.0
42	Wholesale Trade	0.25
44-45	Retail Trade	0.67
48-49	Transportation and Warehousing	1.25
51	Information	0.25
52	Finance and Insurance	0.67
53	Real Estate Rental and Leasing	0.50
54	Professional, Scientific, and Technical Services	0.50
55	Management of Companies and Enterprises	0.50
56	Administrative and Support and Waste Management and Remediation Services	0.80
61	Educational Services	0.33
62	Health Care and Social Assistance	2.85
71	Arts, Entertainment, and Recreation	0.50
72	Accommodation and Food Services	0.78
81	Other Services (except Public Administration)	0.67

Supplementary Table 3. Adjustment of mean work interactions based on Washington State Departments of Health and Labor Statistics[2].

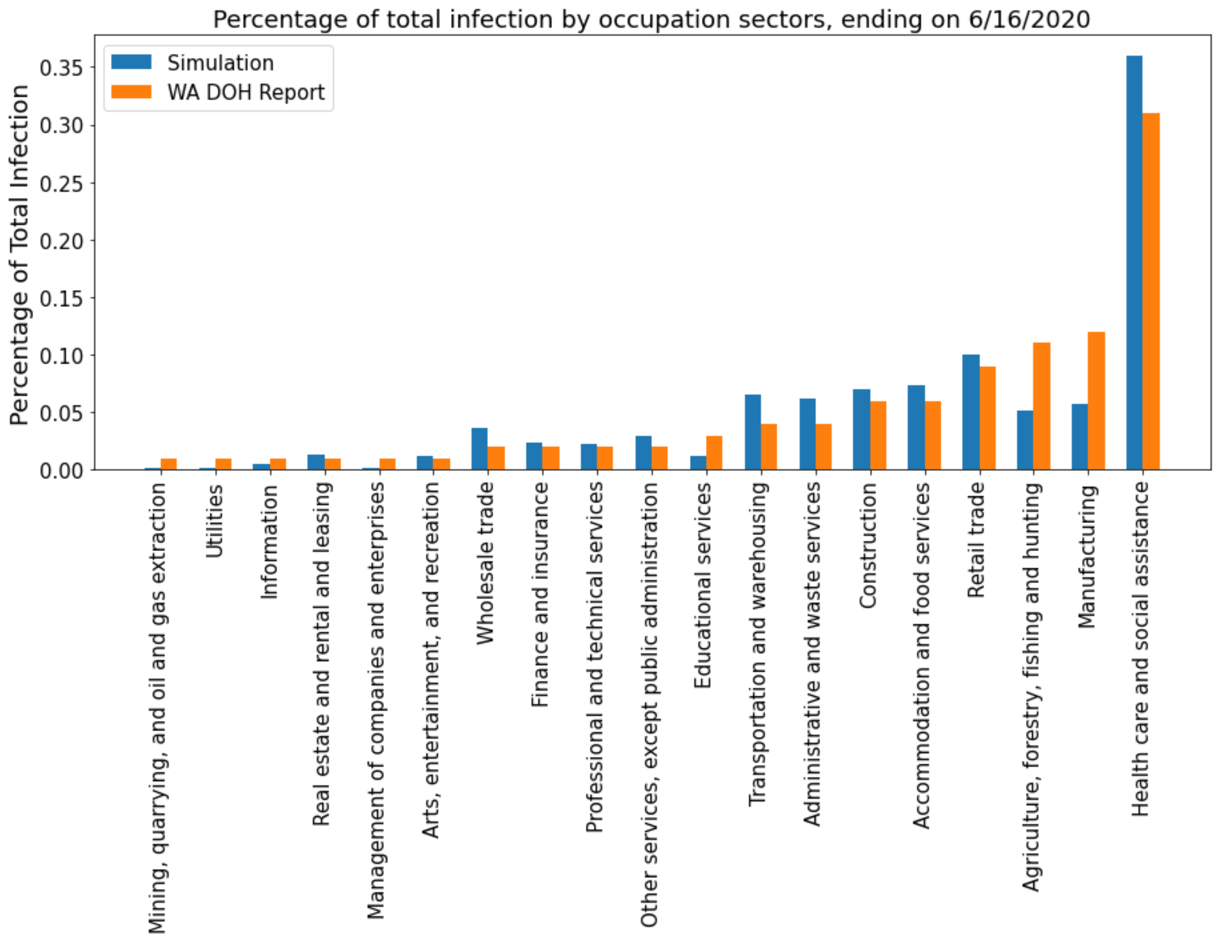

Supplementary Fig. 3. The percentage of total infected cases by occupation sector as reported by the Occupation Industry Report[2] by Washington State Department of Health (state-wide) vs. the OpenABM-Covid19 simulation across King, Pierce, and Snohomish counties.

Sensitivity analysis of detected exposure fraction

The detected exposure fraction is the proportion of infection-spreading interactions that are detected by the app and trigger exposure notifications. Our analysis shows that the

effectiveness of a digital exposure system is impacted by this parameter, especially at the moderate adoption rates (Supplementary Fig. 4, 5). In particular, changes in the detected exposure fraction at low levels significantly affects the rate of new infections across all adoption rates. Increasing adoption is sufficient to counterbalance the effects of lower detection fractions, and conversely, increasing detection fractions improves the performance of exposure notification under lower adoption scenarios (Supplementary Fig. 5).

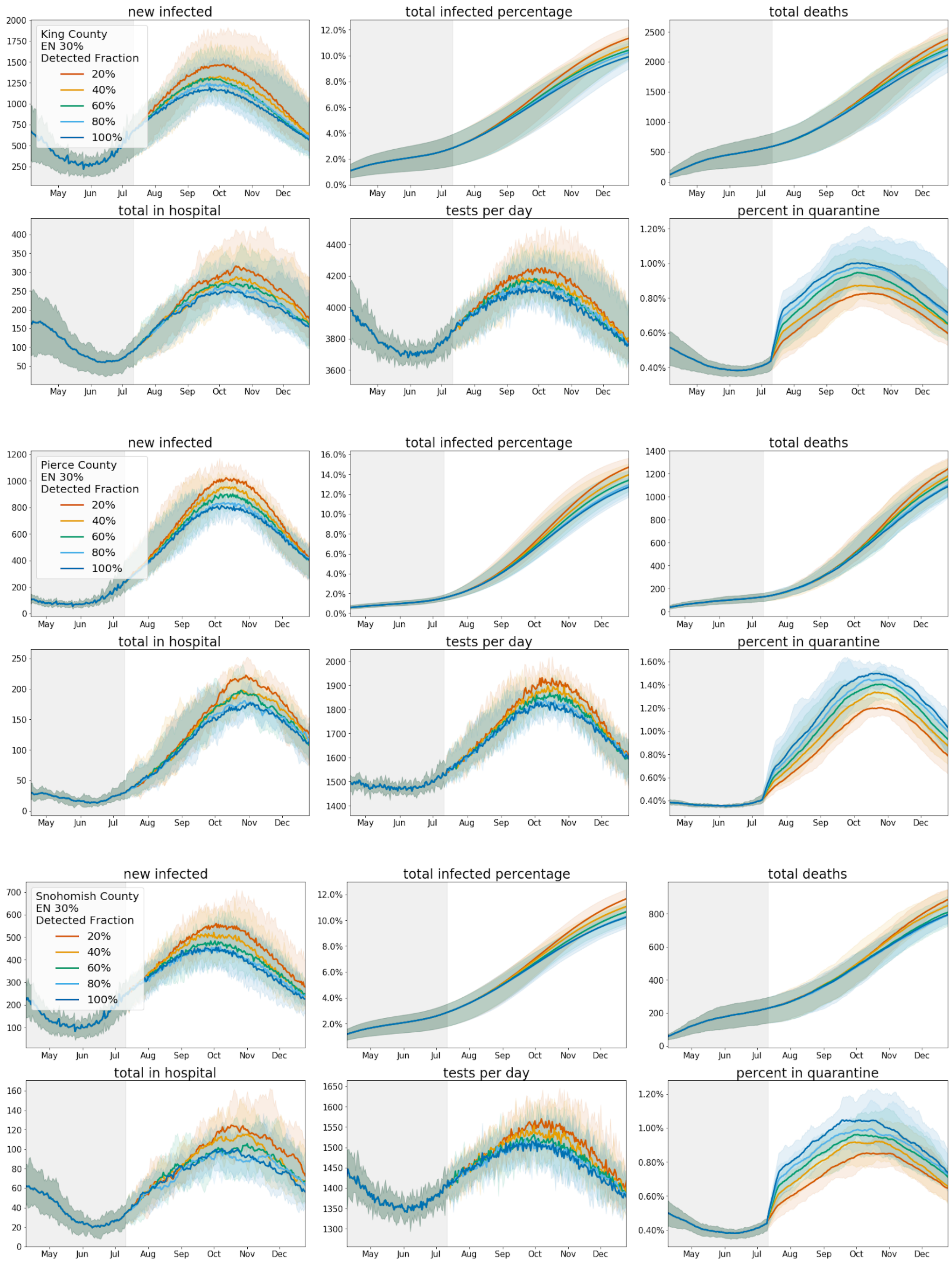
new infected
King County
EN 30%
Detected Fraction
20%
40%
60%
80%
100%
total infected percentage
total deaths
total in hospital
tests per day
percent in quarantine
May
Jun
Jul
Aug
Sep
Oct
Nov
Dec
new infected
Pierce County
EN 30%
Detected Fraction
20%
40%
60%
80%
100%
total infected percentage
total deaths
total in hospital
tests per day
percent in quarantine
new infected
Snohomish County
EN 30%
Detected Fraction
20%
40%
60%
80%
100%
total infected percentage
total deaths
total in hospital
tests per day
percent in quarantine

Supplementary Figure 4. Result of varying the detected exposure fraction at 40% digital exposure notification adoption on the epidemic in King, Pierce, and Snohomish counties.

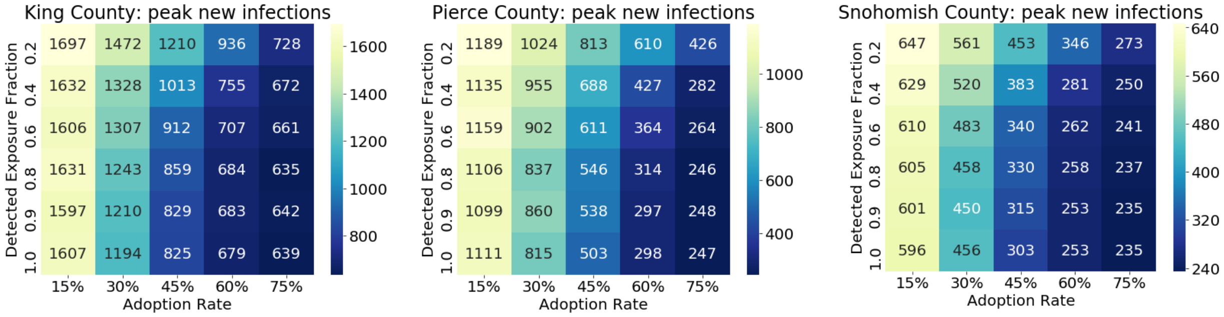

Supplementary Figure 5. The peak new infections after the baseline period of the simulation as a result of varying the detected exposure fraction and digital exposure notification adoption in King, Pierce, and Snohomish counties.

Model calibration

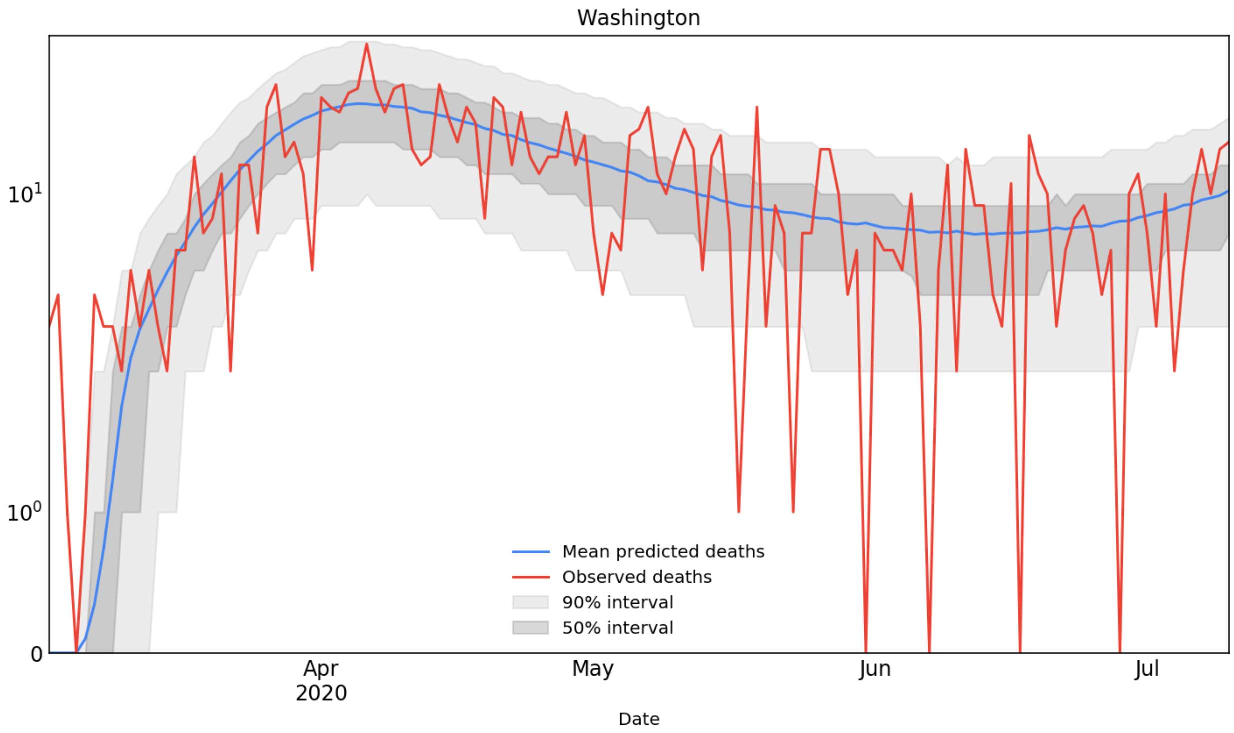

Supplementary Fig. 6. A Bayesian SEIR model fit to Washington state epidemiological data allowing infection rate to vary as a function of human mobility and a latent changepoint to account for unobserved changes in human behavior. See Liu et al.[1] for more detail on the methodology.

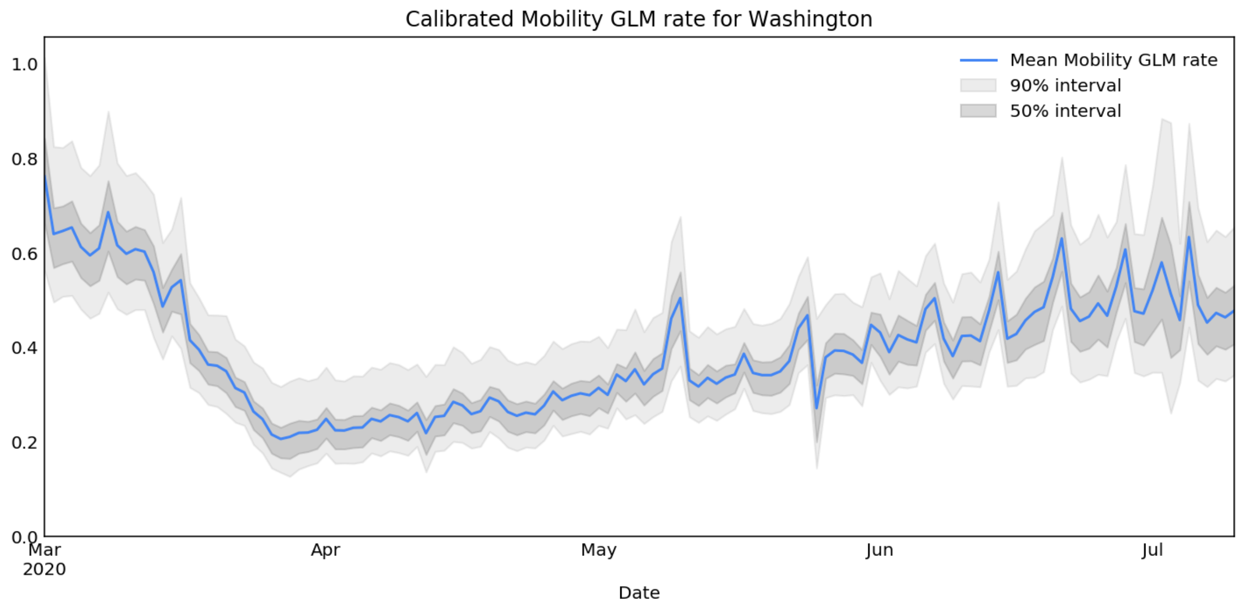

Supplementary Fig. 7. The coefficients from the a generalizable linear model (GLM) fit as part of the above Bayesian SEIR model to predict the time-varying infection rate as a function of aggregated and anonymized mobility data from the Community Mobility Reports. The learned coefficients are used to scale the number of synthetic agent interactions in the random and occupation networks in the OpenABM-Covid19 model.

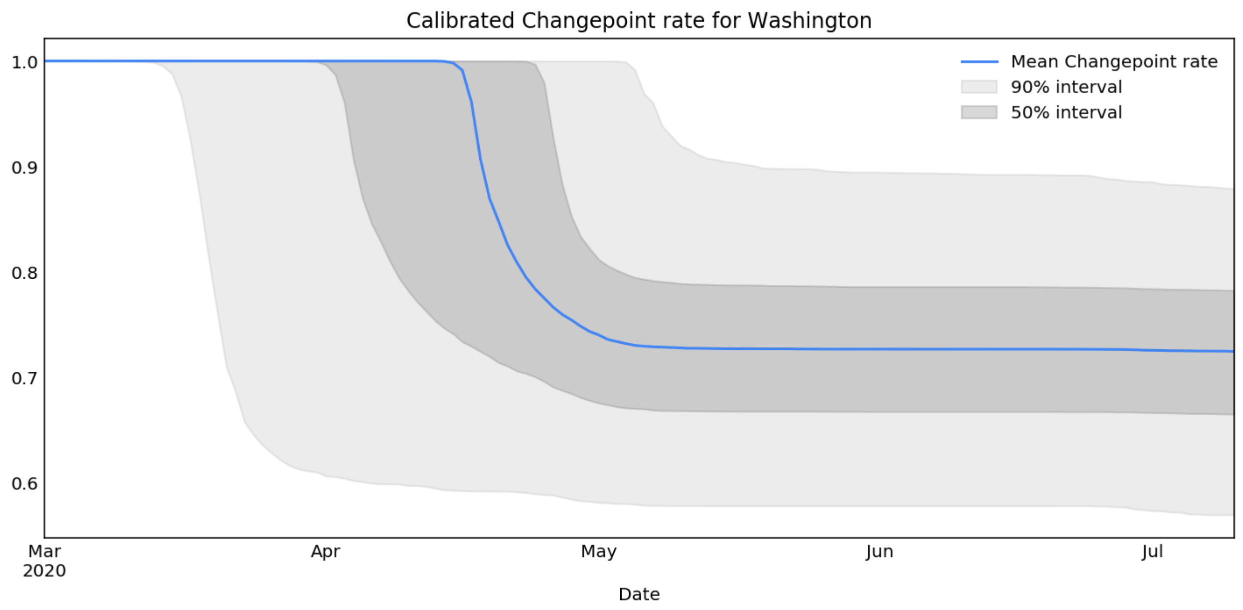

Supplementary Fig. 8. The coefficients of a latent changepoint, modeled as a negative sigmoid, fit as part of the Bayesian SEIR model to predict the time-varying infection rate. The learned coefficients are used to scale the time-dependent infectious rate in the OpenABM-Covid19 model.

County	Initial Infectious Rate	Seeding date (date when the county reaches 30 infections)
King	5.02	02/05/2020
Pierce	5.22	02/16/2020
Snohomish	5.18	02/12/2020

Supplementary Table 4. The initial infectious rate and seeding date for each county, computed via an exhaustive grid search where OpenABM-Covid19 outputs best match COVID-19 mortality from epidemiological data in the county.

References

1. Liu, L. *et al.* Estimating the Changing Infection Rate of COVID-19 Using Bayesian Models of Mobility. https://www.medrxiv.org/content/10.1101/2020.08.06.20169664v1 (2020).
2. Washington State Department of Health (DOH) and the Department of Labor & Industries (L&I). COVID-19 Confirmed Cases by Occupation and Industry. (2020).
